# A metagenomic glimpse into the gut of wild and domestic animals: Quantification of antimicrobial resistance and more

**DOI:** 10.1371/journal.pone.0242987

**Published:** 2020-12-03

**Authors:** Magdalena Skarżyńska, Pimlapas Leekitcharoenphon, Rene S. Hendriksen, Frank M. Aarestrup, Dariusz Wasyl

**Affiliations:** 1 Department of Microbiology, National Veterinary Research Institute, Puławy, Poland; 2 National Food Institute, WHO Collaborating Centre for Antimicrobial Resistance in Foodborne Pathogens, Food and Agriculture Organization Reference Laboratory for Antimicrobial Resistance, and European Union Reference Laboratory for Antimicrobial Resistance, Technical University of Denmark, Kgs. Lyngby, Denmark; 3 Department of Omics Analyses, National Veterinary Research Institute, Puławy, Poland; Nitte University, INDIA

## Abstract

Antimicrobial resistance (AMR) in bacteria is a complex subject, why one need to look at this phenomenon from a wider and holistic perspective. The extensive use of the same antimicrobial classes in human and veterinary medicine as well as horticulture is one of the main drivers for the AMR selection. Here, we applied shotgun metagenomics to investigate the AMR epidemiology in several animal species including farm animals, which are often exposed to antimicrobial treatment opposed to an unique set of wild animals that seems not to be subjected to antimicrobial pressure. The comparison of the domestic and wild animals allowed to investigate the possible anthropogenic impact on AMR spread. Inclusion of animals with different feeding behaviors (carnivores, omnivores) enabled to further assess which AMR genes that thrives within the food chain. We tested fecal samples not only of intensively produced chickens, turkeys, and pigs, but also of wild animals such as wild boars, red foxes, and rodents. A multi-directional approach mapping obtained sequences to several databases provided insight into the occurrence of the different AMR genes. The method applied enabled also analysis of other factors that may influence AMR of intestinal microbiome such as diet. Our findings confirmed higher levels of AMR in farm animals than in wildlife. The results also revealed the potential of wildlife in the AMR dissemination. Particularly in red foxes, we found evidence of several AMR genes conferring resistance to critically important antimicrobials like quinolones and cephalosporins. In contrast, the lowest abundance of AMR was observed in rodents originating from natural environment with presumed limited exposure to antimicrobials. Shotgun metagenomics enabled us to demonstrate that discrepancies between AMR profiles found in the intestinal microbiome of various animals probably resulted from the different antimicrobial exposure, habitats, and behavior of the tested animal species.

## Introduction

The emergence of antimicrobial resistant (AMR) bacteria is one of the most important public health challenges worldwide [1]. Estimates assume that AMR annually triggers more than 700,000 deaths worldwide [2, 3]. In 2015, infections caused by multidrug-resistant bacteria have led to 33,000 deaths in the European Union (EU) and the European Economic Area (EEA) [4]. Recent report of Centers for Disease Control and Prevention points up to 35,000 fatal cases per year due to AMR in the United States [5]. Estimated attributable AMR costs in the EU alone reach 1.5 billion EUR annually [2]. The severe economic consequences encompass also productivity losses in agriculture and animal production [2, 3].

The wide use of similar antimicrobials in human and veterinary medicine contributes to the selection of AMR bacteria and their spread in nature. Thus, the environment polluted with municipal sewage, manure and slurry becomes a reservoir of AMR genes and a risk element in its further transmission [6]. Animals remain a significant vector of AMR determinants in the environment [7, 8]. In Poland, the frequent occurrence of AMR in bacteria isolated from slaughter animals and from food of animal origin is confirmed by the results of AMR monitoring programs [9–11] and studies on AMR in bacteria from wild animals [7, 12]. AMR in commensal intestinal flora of animals might have consequences for the human population: transmission of AMR genes from that reservoir to pathogens such as *Salmonella* might threaten public health through food of animal origin or direct contact with an animal or with animal husbandry facilities [8, 9, 13].

Several aspects have an impact on the occurrence and spread of AMR in animals. Animal behavior influences potential exposure to the acquisition of resistant bacteria. Differences in diet may alter the intestinal microbiome. Feed may be a source of compounds affecting gut resistome and serve as a vector of AMR determinants [14, 15]. Bearing in mind the complexity and scale of AMR and the number of factors driving the increase in AMR, it is reasonable to apply a multidirectional approach analyzing the impact of diet and animal behavior on the occurrence of the phenomenon. A metagenomic approach using shotgun sequencing provides such possibility and it is essential in the context of AMR genes within a mixed bacterial population [16]. Notably considering that AMR genes are often located within mobile genetic elements (e.g. plasmids, transposons, integrons) that enable their horizontal transfer, even between unrelated bacterial species [17].

For effective combat of increasing bacterial AMR in times of intensification of animal production, the growing popularity of game meat and expansion of the human population, it is particularly important to assess the role of different animal species in AMR dissemination [18].

Herewith we used a powerful shotgun metagenomic tool to prove the hypothesis that diverse antimicrobial exposure, habitats, and feeding behavior of different animal species lead to discrepancies between AMR profiles found in their intestinal microbiome.

To investigate the AMR epidemiology we selected intensively produced farm animals: chickens, turkeys, and pigs, which are often exposed to antimicrobial treatment opposed to a unique set of wild animals such as wild boars, red foxes, and rodents that seems not to be subjected to antimicrobial pressure.

The rationale for examining poultry was short fattening time/life span and AMR group treatment practiced during breeding. The inclusion of pigs and wild boars representing *Sus scrofa* allowed to compare the same species living in free and farmed conditions. The study of red foxes (predators) and rodents as their presumed prey was another benefit that allowed us to look into feasible AMR genes flow within the trophic chain.

Our objective was to investigate and quantify the scale of the AMR phenomenon in several species of domestic and wild animals. We applied shotgun metagenomics of total DNA isolated from intestinal content of animals to explore the abundance of different resistance genes, and to examine bacterial and plasmid composition. We also looked at the possible diet of selected animals in the context of AMR spread.

## Materials and methods

### Sample collection

A total of 60 samples of intestinal content from different farm and wild animals originating from 2016 and 2018 were selected among the numerous samples available at the National Reference Laboratory for Antimicrobial Resistance (NRL) Poland. Samples included farm animals therein extensively produced poultry: chicken broilers (*Gallus gallus*, n = 10), turkeys (*Meleagris gallopavo*, n = 10), pigs (*Sus scrofa*, n = 10) and also wild animals represented by wild boars (*Sus scrofa*, n = 10), red foxes (*Vulpes vulpes*, n = 10) and rodents: eight forest mice (*Apodemus flavicollis*), one field mouse (*Apodemus agrarius*) and one field vole (*Microtus arvalis*). All samples were derived from healthy animals. In case of farm species, samples were collected close to slaughter and constituted fraction of samples tested within the EU-monitoring (2017–2018). Wildlife samples came from animals covered by rabies and *Leptospira* control programs.

Red fox samples were collected from animals hunted during nine events in 2018. Wild boars feces came from animals shot during ten hunts between 2017 and 2018. No ethical approval was required for collection of samples from slaughter animals, red foxes and wild boars, yet all procedures were in accordance with Polish law and The Act on the protection of animals of August 21th, 1997 (Journal of Laws 1997 No. 111 item 724 as amended). Slaughter animal samples were collected within 2013/652/EU: Commission Implementing Decision of 12 November 2013 on the monitoring and reporting of antimicrobial resistance in zoonotic and commensal bacteria. Red fox and wild boars samples collected under the Polish Regulation Ordinance of the Minister of Agriculture and Rural Development of December 17, 2004 regarding the definition of disease entities, the manner of conducting control and the scope of control tests of animal infections.

Rodents were captured during 2016 and 2017 for the purpose of grant on *Leptospira*. All procedures were carried out according to the ethical standards for the use of animal samples and were approved by the Local Ethics Committee for Animal Experimentation in Lublin, Poland (Resolution No. 30/2016). Animals were caught in their natural foraging areas (forests and meadows) using Sherman traps. Live animals were transported to the laboratory and euthanized on the same day with Isofluranum. The anesthetic was dosed according to the manufacturer's recommendations. All efforts were made to minimize animals suffering.

Upon arrival at NRL samples were frozen and stored at −80°C until further processing in autumn 2018.

### DNA extraction and pooling

All samples were only thawed once just before DNA extraction. Total DNA was extracted from each sample with a QIAamp Fast DNA Stool Mini Kit (product number 51604, Qiagen) according to a published protocol [19] with modifications as described previously [20]. Following DNA extraction, all samples were measured with NanoDrop One (Thermo Scientific) for yield and as a purity check, and with Qubit Fluorimeter (Invitrogen). Ten samples from each species or order (in case of rodents) were subsequently pooled based on quantitative fluorimetric results, to obtain an equal proportional representation of each individual in a pool.

### Library preparation and sequencing

DNA was shipped on dry ice for library preparation and sequencing at the National Food Institute, Technical University of Denmark. DNA libraries prepared with a Nextera Library Preparation Kit (Illumina) were subsequently sequenced with the NextSeq platform (Illumina), using 2 × 150 paired-end sequencing per flow cell. A high output flow cell was used with a triple-capacity FC-404-2004 NextSeq 500/550 High Output v2 kit (300 cycles).The reads were deposited at the European Nucleotide Archive (ENA) (http://www.ebi.ac.uk/ena/data/view/PRJEB40824).

### Bioinformatics processing

BBduk (BBMap software) was applied for raw read trimming [21] and BWA-MEM algorithm was exploited for removing the phiX174 internal sequencing control [22]. Trimmed paired-end reads from each metagenomic sample were mapped using the MGmapper tool [23] against database of acquired AMR genes–ResFinder (version 20180921) [24] and databases containing genome sequence data from GenBank (http://www.ncbi.nlm.nih.gov/genbank/). The selected databases were mapped in the following order: ResFinder and Plasmid (version 20180226) in option fullmode, and Bacteria (version 20180226), Bacteria_draft (version 20180226), Vertebrates_mammals (version 20180306), Vertebrates_other (version 20180306), Invertebrates (version 20180306), Plant (version 20180306) with bestmode approach.

Bestmode mapping, based on the highest alignment score, assigned the read-pair to only one reference sequence in one of all specified bestmode databases. In case of equal alignment scores the read-pair was assigned to the database defined as first. Fullmode option as previously described was applied for AMR genes and plasmid databases to enable read mapping to multiple databases [23].

### Data analysis

The results of ResFinder read mapping for individual genes were aggregated to clusters based on 90% identity as described previously [20]. Based on raw read counts, the relative abundances of AMR genes, plasmids and bacteria were estimated. Calculations accounted for gene length and the number of bacterial reads was determined as fragments per kilo base reference per million bacterial fragments (FPKM) [6].

To validate our data, AMR results obtained for broiler chickens and pigs were compared to the data of Polish broiler and pig samples included in the EU-funded EFFORT project [20]. In the cases of plant and animal read counts, relative abundances were calculated taking into account the number of reads mapped to a specific taxonomic group per total number of reads in the sample multiplied by 10^6^ and the results presented as reads per million (RPM).

Relative abundance values were visualized in heat maps. Reads mapped to Vertebrates_mammals, Vertebrates_other, Invertebrates and Plants databases were considered as potential diet components of the tested animals. Examples of crops, fodder plants, wild plants, different animal species and insects were selected for the analysis. As for rodents, reads assigned to the *Muridae* and *Soricidae* families were considered the host material, similarly in poultry samples reads mapped to the *Anatidae* and *Phasianidae* families were disregarded.

The limitation of the method is that mapping to highly homologous sequences might result that the reads are assigned to related species. To reduce the bias of possible incorrect mapping when one genus was represented by several species, the sums of those reads were shown on heat maps.

Analysis and visualization of results on graphs and heat maps were carried out in the open source RStudio 3.5.3 version for Windows (https://www.rproject.org/) using the library(vegan), library(pheatmap), library(ggplot2), library(reshape2), library(RColorBrewer), library(plyr), and library(grid) packages. The exception was Fig 5 prepared in Excel 2016 (Microsoft Office). The diversity of bacterial species and AMR genes noted in samples was measured with the Shannon and Simpson’s diversity indexes. The Chao1 richness was also estimated.

## Results

### Resistome diversity

**Fig 1:**
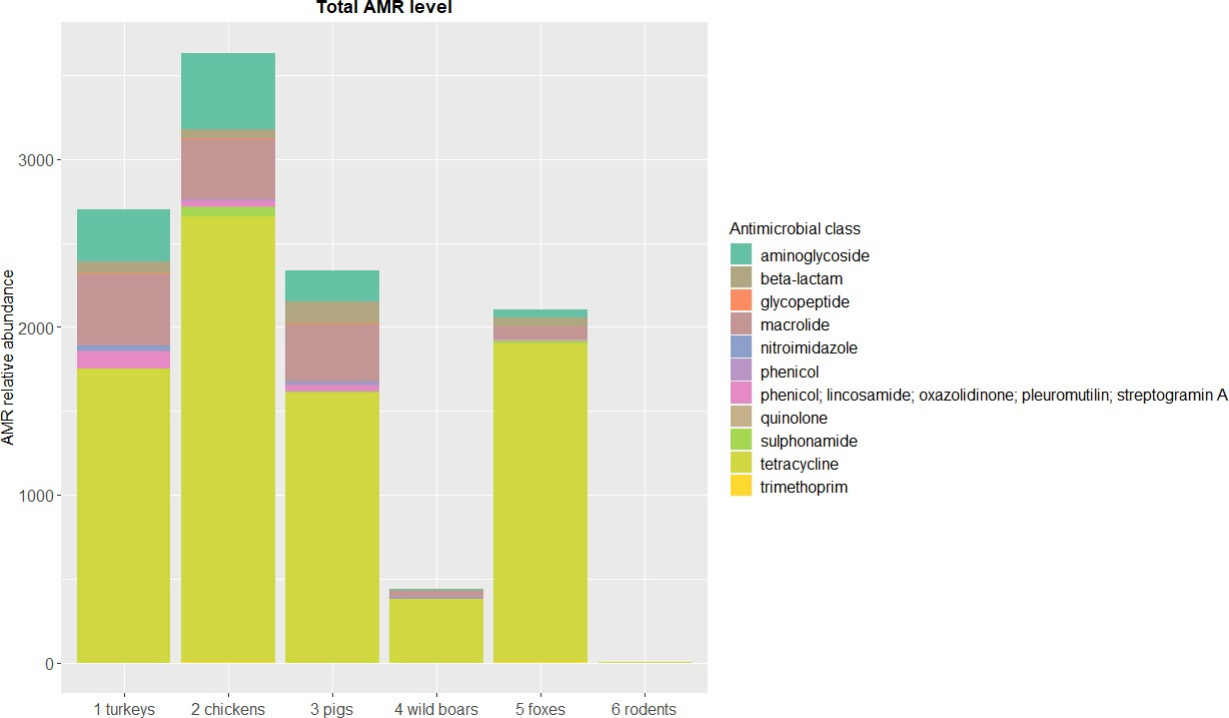
Total level of antimicrobial resistance genes by drug class and animal source. Stacked column chart with relative abundances (FPKM) of AMR genes aggregated to corresponding drug classes (y-axis) by sample (x-axis). The height of each bar chart relates to the relative AMR gene abundances in a sample.

**Fig 2:**
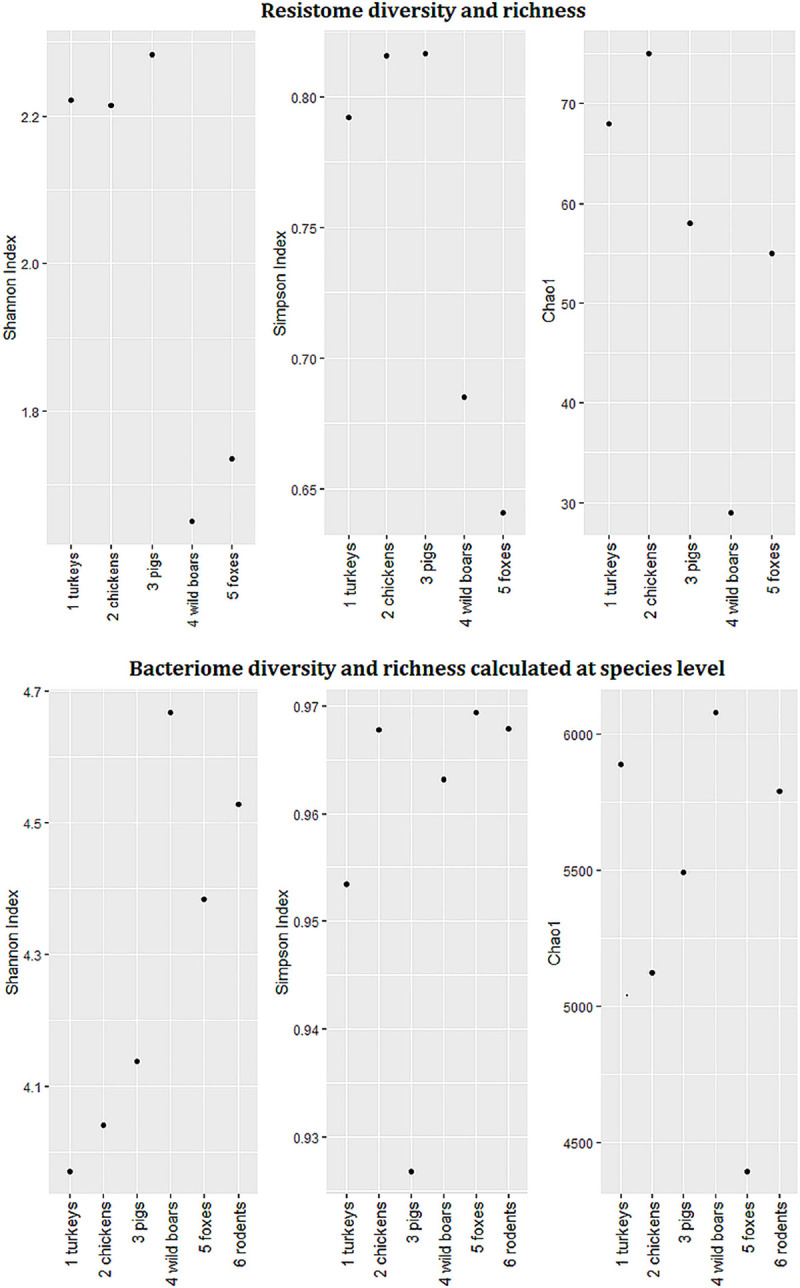
Resistome and bacteriome diversity and richness. Shannon, Simpson diversity indexes and Chao1- richness calculated from the read counts.

The entire sequencing dataset yielded on average over 21.6 billion base pairs (bp) per sample. From over nine hundred million reads (ranging from ~111 to ~196 million reads per sample) 0.03% were attributed to AMR genes (S1 Table). In total, we identified 117 different AMR gene clusters covering 386 AMR gene variants. The number of AMR genes differed between tested animals, and in general, higher levels of AMR genes were observed in food-producing animals than in wildlife. The highest abundance of AMR determinants of all the tested animals was observed in chickens (75 AMR gene clusters, over 3,600 FPKM). In wildlife, foxes displayed the largest number of reads assigned to AMR genes (55 gene clusters, above 2,100 FPKM), but in rodents less than three FPKM reads (five gene clusters) mapped to the ResFinder database (Fig 1, S2 Table). The observed diversity of AMR determinants was higher in farm animals (Fig 2). Due to the small number of reads mapped to the ResFinder database, we excluded rodent sample from calculations of AMR gene diversity and richness indexes.

**Fig 3:**
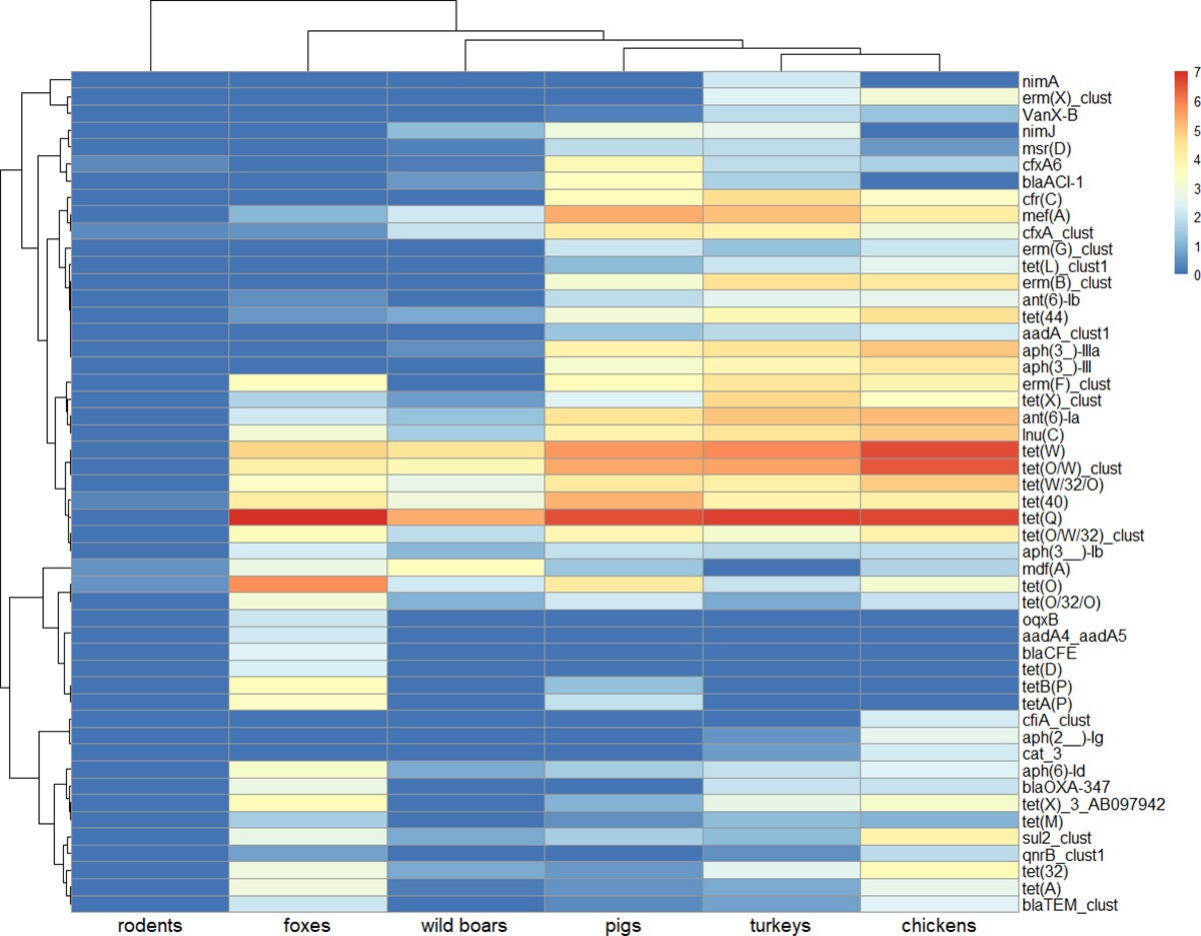
The most abundant antimicrobial resistance genes by animal source. AMR genes abundances heat map based on log transformed relative abundances—FPKM values. Colors scale from red (high abundance) to blue (low abundance) represent log transformed relative abundance. Dark blue (0 on a scale) means no resistance detected.

Of all AMR classes assessed, tetracycline resistance dominated (Fig 1, S2 Table) with *tet*(Q) being the most abundant gene in all tested animals except rodents. The *tet*(X) gene that confers resistance to tetracyclines and tigecycline was also noted (Fig 3). Depending on animal species, macrolide (turkeys, pigs, wild boars, and foxes), aminoglycoside (chickens) or beta-lactam (rodents) resistances were the second most abundant (Fig 1, S2 Table). Macrolide resistance determinants were dominated by *mef*(A), *lnu*(C), and *erm*(B) in farm species while *mdf*(A) was more abundant in foxes and wild boars (Fig 3). Aminoglycoside resistance predominated in farm animals, and *ant(6)-Ia*, *aph(3′)-III*, and *aph(3′)-IIIa* genes prevailed. Determinants encoding AMR towards beta-lactams, e.g. *cfxA6* or *bla*_ACI_ were more often found in pigs and were seen at comparable levels in both tested poultry species e.g. *cfxA*, *bla*_OXA-347_. It is worth emphasizing that *bla*_OXA-347_ prevailed in foxes and a few other beta-lactam genes encoding AmpC type β-lactamases, e.g. *bla*_CFE_, *bla*_DHA_, *bla*_CMY_ (the blaCMY–blaBIL–blaLAT cluster) were unique to this species (Figs 1 and 3, S2 Table). *cfr*(C) gene conferring cross-resistance to phenicols, lincosamides, pleuromutilins, streptogramin A and oxazolidinones was only found in farm animals (Fig 3). AMR towards other classes of antimicrobials were less abundant. Among them, quinolone resistance was more often detected in foxes and chickens, while no such resistance was noted in pigs or rodents. The specific genes corresponding to this AMR profile were *qnr*B and *qnr*S in poultry and *oqx*A, *oqx*B, and *qnr*B in foxes. Glycopeptide resistance determinants were observed in all farm species and wild boars. No plasmid-mediated colistin resistance was detected in any of the tested animals (Figs 1 and 3, S2 Table).

Dendrogram for genes was clustered on Pearson correlation coefficients, whereas for samples it was based on the Bray-Curtis dissimilarity indexes. Complete linkage clustering was applied for dendrograms. Heat map presents the 50 most abundant determinants but all resistance genes found in samples were included in computations.

### Plasmid evidence

**Fig 4:**
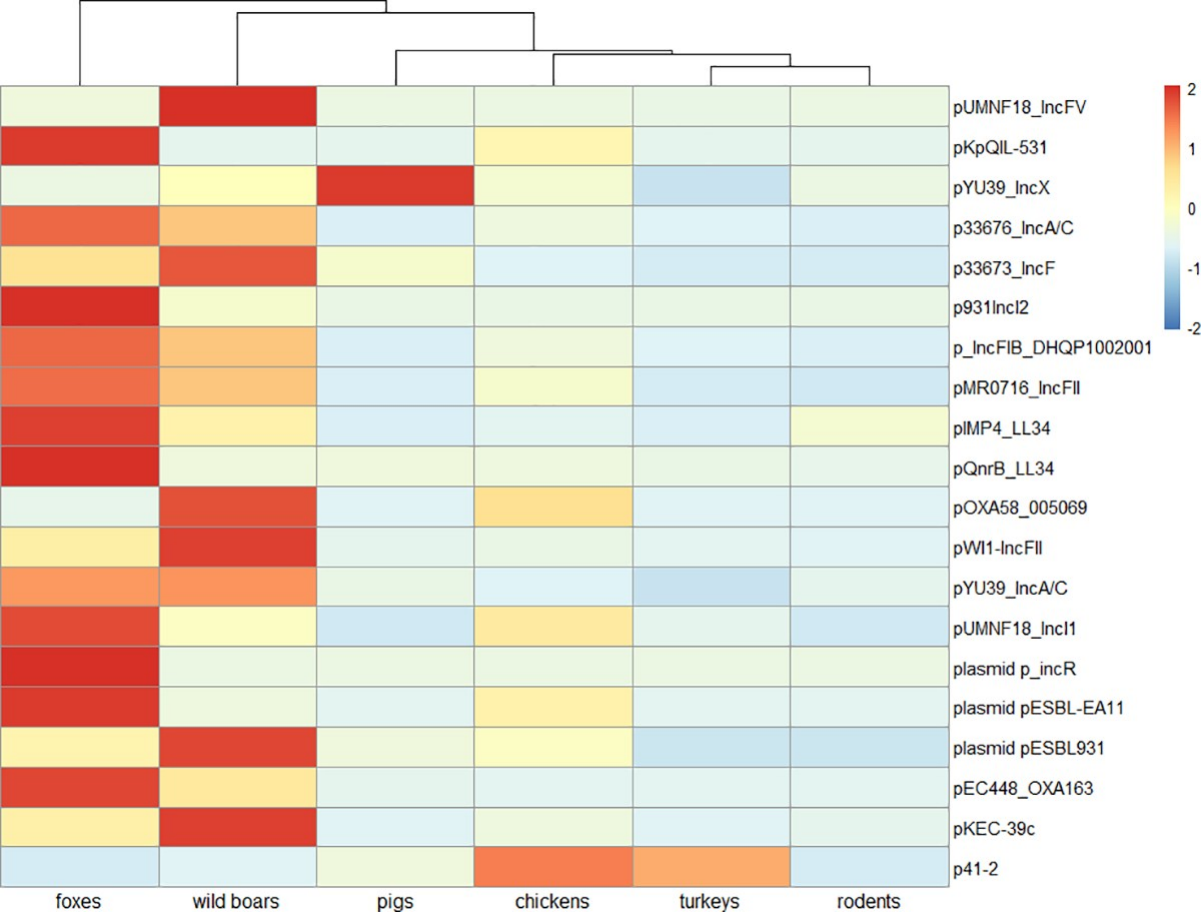
Plasmids possibly involved in resistance transfer. Heat map based on plasmids relative abundances with Z-score scaling. Samples with high relative abundances get positive values (red color) and those with relatively low get negative values (blue colors). Complete-linkage clustering of Euclidian distances was applied for clustering the samples.

The highest entire plasmid content were observed in wild boars and foxes (over 990 FPKM and 650 FPKM, respectively) in comparison to other animals tested. Lower levels were found in poultry species (over 590 FPKM in chickens and 480 FPKM in turkeys) and in rodents (over 360 FPKM). Interestingly, pigs had the least observed number of reads mapped to the plasmid database with less than 250 FPKM (S2 Table). Evidence of plasmids associated with AMR transfer was noted within plasmid profiles of tested animals. Among those, traces of plasmids belonging to incompatibility groups IncF, IncA/C, IncI, IncR were found. The particular were more abundant in wild boars and foxes. Traces of IncX predominated in pigs. Occurrence of plasmids possibly involved in AMR transmission is depictured in Fig 4.

### Bacterial composition

**Fig 5:**
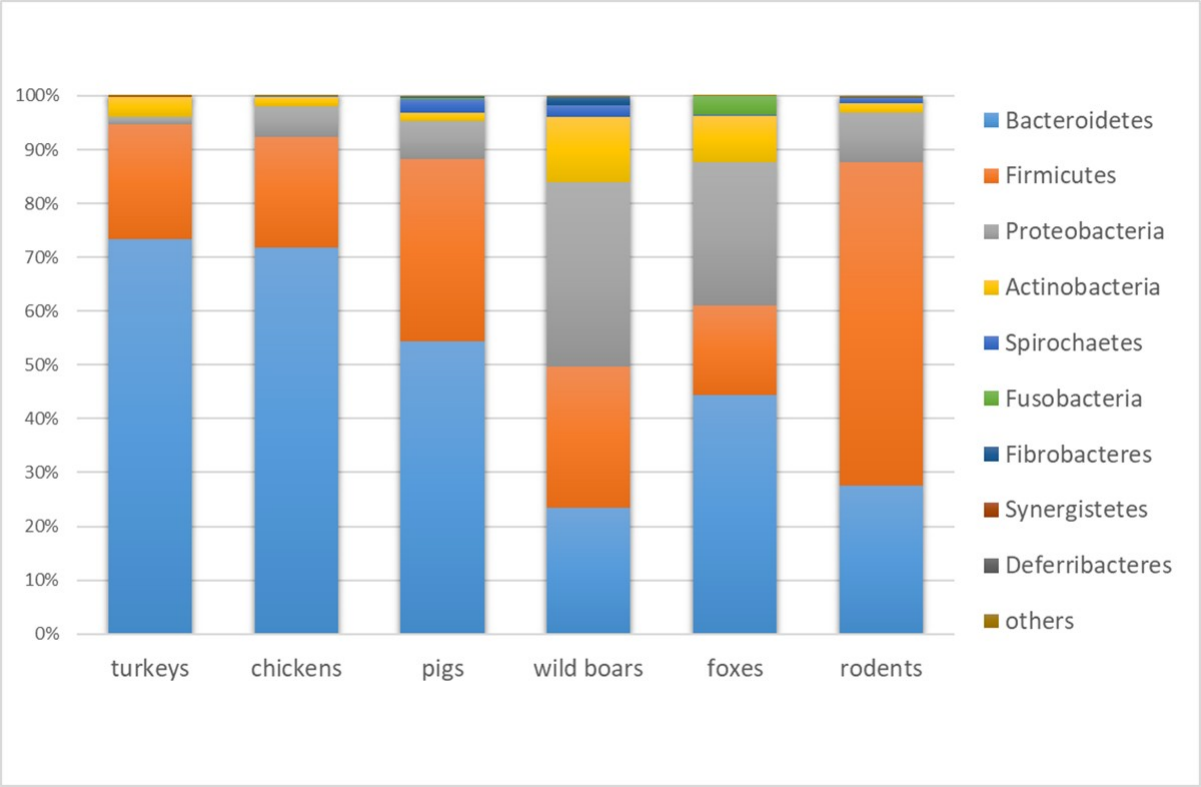
Bacterial composition at phylum level by animal source. Stacked column chart with relative abundances of the most abundant bacterial phyla based on relative abundances.

**Fig 6:**
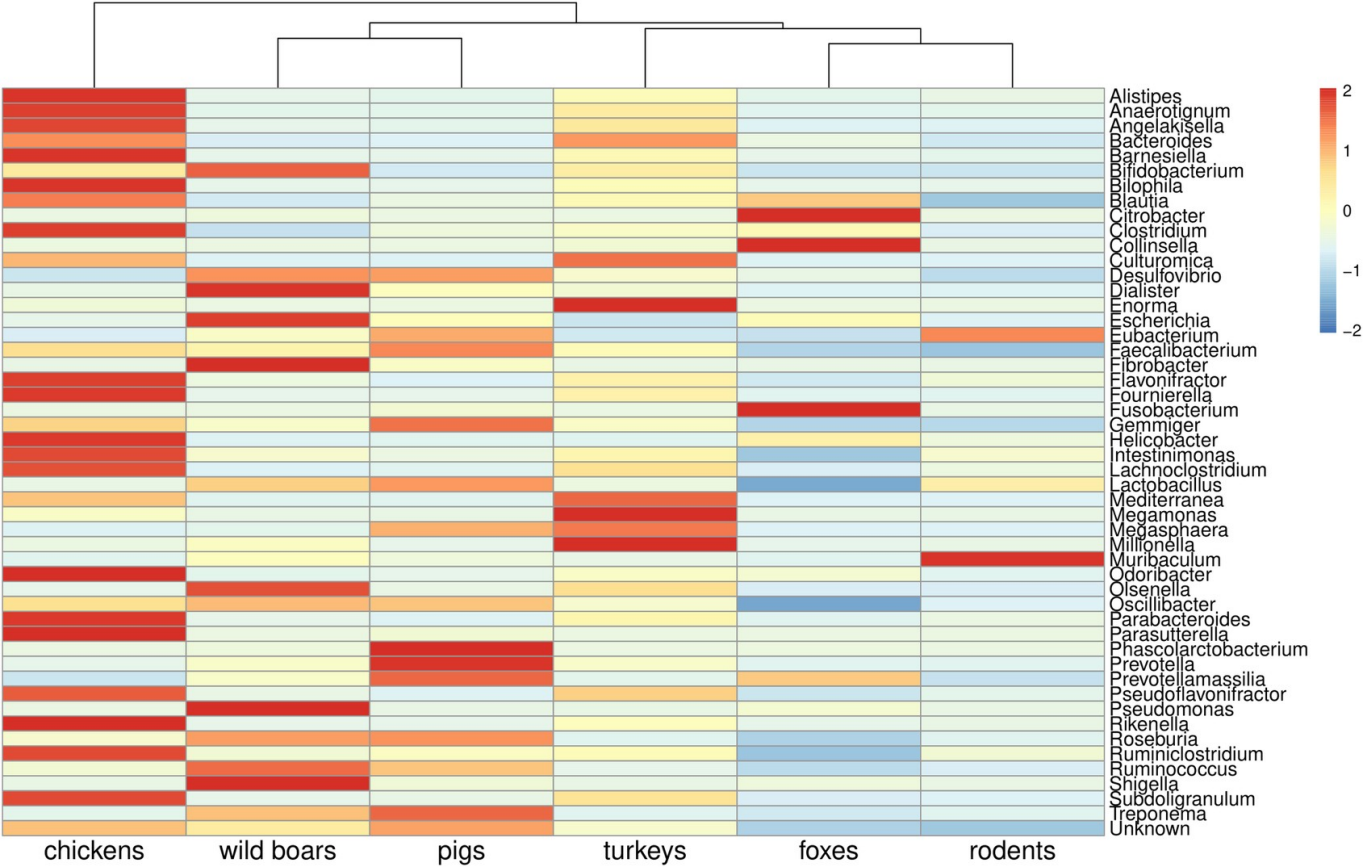
Bacterial composition at genera level by animal source. Heat map presents the 50 most abundant bacterial genera based on relative abundances values with Z-score scaling. Samples with high relative abundances get positive values (red color) and those with relatively low get negative values (blue colors). Complete-linkage clustering of Euclidian distances was applied for clustering the samples.

**Fig 7:**
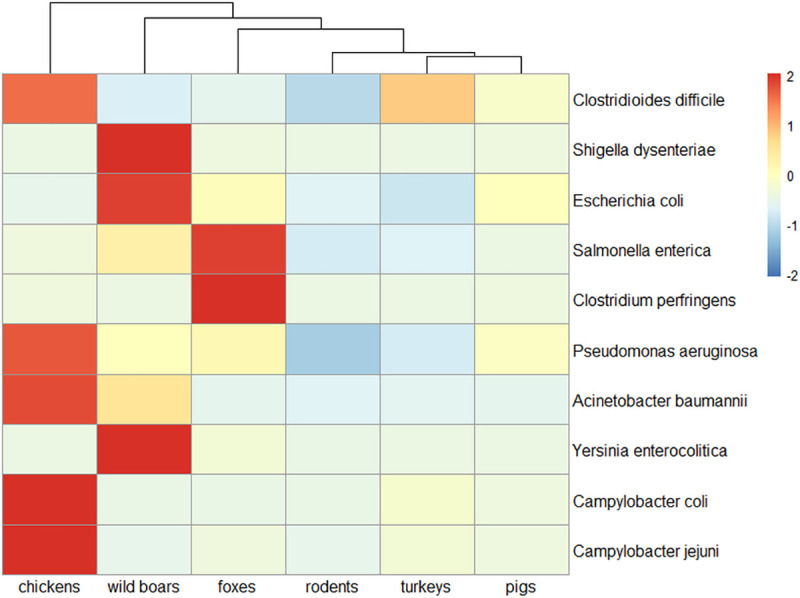
Pathogens occurrence by source animal. Heat map of selected bacterial pathogens based on relative abundances values with Z-score scaling. Samples with high relative abundances get positive values (red color) and those with relatively low get negative values (blue colors). Complete-linkage clustering of Euclidian distances was applied for clustering the samples.

Reads assigned to bacterial genomes constituted 8.05% of all reads (S1 Table). Differences in bacterial composition were observed, and the bacteriome was generally more diverse in wild animals (Fig 2). Bacteria belonging to *Bacteroidetes*, *Firmicutes*, *Proteobacteria*, and *Actinobacteria* were the most abundant, but discrepancies in specific phyla contribution were observed in different animals. The highest level of *Bacteroidetes* was found in farm animals (more than 50% prevalence in pigs and over 70% in poultry species) (Fig 5). The rodent microbiome was dominated by *Firmicutes*, whereas in wild boars and foxes, high levels of *Proteobacteria* and *Actinobacteria* were observed. Among 1,936 detected bacterial genera, including those typical in fecal samples, e.g. *Bacteroides*, *Escherichia*, and *Faecalibacterium*, there were also bacteria with zoonotic potential observed. Evidence of *Salmonella* in foxes, *Yersinia* and *Shigella* in wild boars and *Campylobacter* in chickens was noted (Figs 6 and 7).

### Diet composition

**Fig 8:**
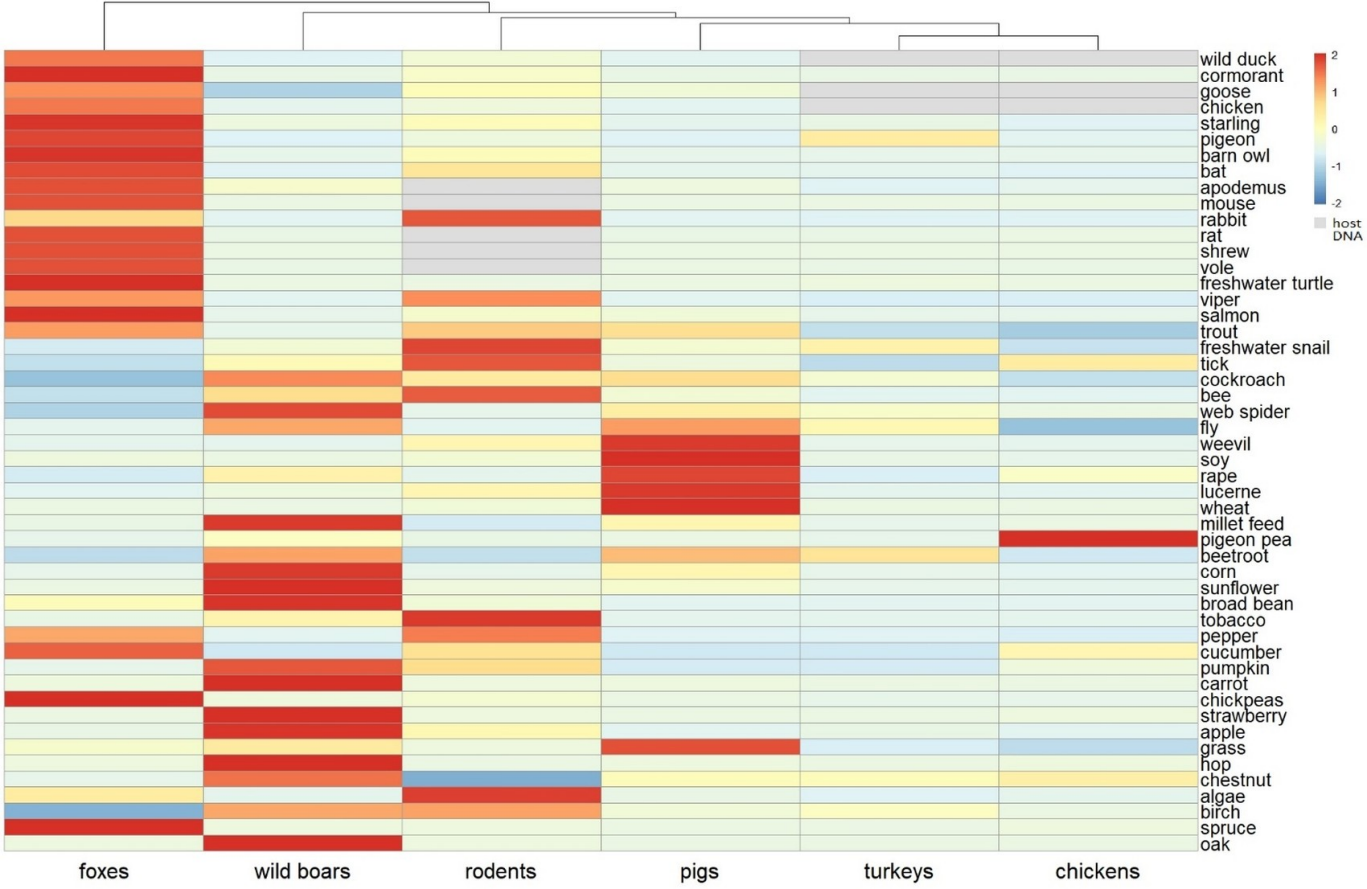
Diet of tested animals. Heat map based on relative abundances (in RPM: reads per million) of selected plants, vertebrates and invertebrates with Z-score scaling. Samples with high relative abundances get positive values (red color) and those with relatively low get negative values (blue colors). Grey color corresponds to host DNA. Complete-linkage clustering of Euclidian distances was applied for clustering the samples.

Reads assigned to plant, vertebrate and invertebrate genomic material represented 0.02%, 20.22% and 0.03% of all reads respectively (S1 Table). Poultry intestines contained traces of fodder plants, e.g. pigeon pea or beetroot. Reads mapped to goose, wild duck, and chicken DNA corresponded to host DNA in the two samples. In pigs, high levels of reads mapped to fodder plants like lucerne, rape, wheat or soya. Insects and fish genetic material were also found in this species. In wild boars, crop plant evidence, e.g. hop, broad bean, pumpkin, beetroot and wild plants like oak or birch was observed. Intestinal contents of foxes contained DNA of wild birds, reptiles, rodents, fish, and traces of fodder and wild plants. In case of rodents tobacco, birch, pepper, and algae dominated plant DNA material, however, DNA of insects was also observed (Fig 8).

## Discussion

Data collection on AMR determinants in animals is crucial to effectively combat resistant bacterial pathogens that may affect consumer health not only *via* food of animal origin but also through direct contact with the animal or animal husbandry environments [9]. Therefore, our objective was to investigate the role of different animal species as possible reservoirs of AMR determinants. Applied shotgun metagenomics enabled to examine animals so different in diet, behavior and habitat in the context of AMR spread. Applying the same methodology to the preparation of samples raised the quality of the results and had a significant impact on the accuracy of the comparison of different animal species. The entire sequencing yield was in accordance with previous metagenomics studies and the amount of data generated was adequate to quantify the AMR gene contents and investigate fecal bacterial composition [6, 20].

Not surprisingly, our findings revealed higher AMR level in domestic animals in comparison to wild. Tetracycline resistance dominated AMR profiles and this probably results from ongoing selective pressure in the environment, as tetracyclines have been the most widely applied antimicrobial class in veterinary medicine and horticulture over decades [25, 26]. According to European Surveillance of Veterinary Antimicrobial Consumption (ESVAC) data only in 2017, tetracyclines constituted 30.4% of entire veterinary antimicrobials sale within 31 European countries [26]. Other AMR genes that prevailed in farm animals belonged to the classes critically important in humans: macrolides, aminoglycosides and beta-lactams. AMR patterns observed in chickens and pigs dominated with above mentioned classes of antimicrobials were consistent with results of the EFFORT project [20]. These results were also compliant with data from other metagenomics studies on chicken and pig microbiomes that indicated specified antimicrobial classes as the frequently abundant [20, 27–31].

In comparison to wild animals, resistomes of domestic species were characterized by greater gene diversity and richness. These results and the overall AMR level in domestic animals would clearly indicate the selective effect of antimicrobial usage (AMU) [26]. High correlations between AMU and AMR were indicated by a previous study [32]. According to ESVAC data, penicillins, tetracyclines and macrolides were listed among the top three antimicrobial classes most often administered for food-producing animals in Poland reaching in 2017 respectively: 54.1, 47.9 and 18.1 in mg per population correction unit (mg/PCU) [26]. Aminoglycosides, relatively less applied in the field (4.7 mg/PCU) occupy the eighth place among drugs administered in Poland [26]. The high level of aminoglycosides AMR might be explained by co-selection with other antimicrobials [26, 33]. Previous research reported development of aminoglycoside resistance as a consequence of chlortetracycline, sulfamethazine, lincomycin and penicillin treatment [34, 35].

Other aspects that may affect the result are indicated by analysis of the potential diet. We found the presence of DNA host material in all of the tested samples and expected diet composition. As predicted, evidence of fodder plants in farm animals, including imported ones, e.g. pigeon pea (*Cajanus cajan*) was noted [36]. In the context of AMR spread discovering traces of insects like flies, weevils, cockroaches in pig samples captured our attention. It was revealed formerly that such insects might serve as vectors of pathogenic bacteria and AMR determinants towards antimicrobials critically important in humans: beta-lactams, quinolones, aminoglycosides, macrolides and others [37–40].

The theory of the selective effect of AMU may be supported by the high read abundances of the *cfr*C gene noted only in farm species. This plasmid-mediated gene determines cross-resistance to phenicols, lincosamides, pleuromutilins, streptogramin A and oxazolidinones, including linezolid, listed as a critically important antimicrobial [41]. The gene was reported in multidrug-resistant *Campylobacter* and *Clostridium difficile* isolates [42, 43]. In this light, the evidence for pathogens we found in poultry indicates a serious threat to public health. We assume that *cfr*C abundance is associated with selection by pleuromutilins. Sales of this antimicrobial class in Poland for food-producing animals in 2017 reached 8.4 mg/PCU [26].

Importantly, our study shown the presence of *tet*(X) gene in all tested animals except rodents and found it in highest abundance in poultry, particularly turkeys. The result is cause for concern, as the gene encodes resistance not only to tetracyclines but also to tigecycline, the last resort antimicrobial against multi‐drug resistant *Enterobacteriaceae* and methicillin‐resistant *Staphylococcus aureus* [41]. The abundance of *tet*(X) in pig and chicken samples were reported formerly [28, 31].

Traces of determinants encoding class D beta-lactamases were noted in poultry. Among them we found *bla*_OXA-347_ gene both in chickens and turkeys. We assume that this result might be an outcome of penicillins or cephalosporins use as carbapenems are prohibited in livestock treatment in Poland. Previously presence of *bla*_OXA-347_ was described in porcine gut microbiome as a result of amoxicillin treatment [44]. In fact, penicillins are the most commonly used for food-producing animals in Poland. Cephalosporins estimate sale in 2017 totaled 0.3 (mg/PCU) [26]. In chickens we noted also chromosomal *cfiA* gene encoding metallo-β-lactamase. Other studies confirmed the gene in *Bacteroides* associated with human infections [45, 46] and our study identified this genus among the most abundant bacterial genera in both tested poultry samples.

Anthropogenic impact on AMR spread might be highlighted by AMR profiles in two species belonging to *Suidae*: wild boars and pigs. High levels of AMR towards tetracycline, beta-lactams, and macrolides in pigs comparing to wild boars indicate the selective effect of antimicrobial classes widely used in pig production sector [47]. Interestingly only in food animals and in wild boars we noted resistance to glycopeptides. Vancomycin resistant enterococci isolated from wild boars were reported in Portugal and Spain [48, 49]. AMR towards vancomycin might be aftermath of the avoparcin administration [50]. This glycopeptide was widely used as a growth promoter in animal production until its ban in 1997 [51]. Glycopeptides resistance as a consequence of co-selection with AMR towards other antimicrobials e.g. macrolides is also possible and this scenario seems to be the more probable [52]. Recent evaluations of erythromycin occurrence in water demonstrated that this macrolide residues are common in the environment [53]. It should be emphasized that significant amounts of antimicrobials excreted as “still-active compounds” that even in sub-therapeutic doses may select for AMR [25]. Hence, it can be assumed that the prevalence of AMR in wildlife might result from the selective pressure of antimicrobials present in organic substances e.g. slurry used as agricultural fertilizers. The detection of crop plants in the intestinal contents of wild boars proves that animals invade farmlands foraging for food and therefore might be exposed to drug residues. Furthermore, the presence of chemicals, e.g. pesticides used in the control of some plant diseases, should also be taken into account as those compounds may also induce AMR [54].

Our analysis revealed a significant AMR level including resistance to drugs highly important for public health in red foxes. Exposure to AMR derives from discarded food and agricultural waste near urban settlements visited by scavenging animals might to some extent explain the AMR level in this species. The results also indicate that apex predatorial red foxes may accumulate resistance determinants form wild birds, small mammals or reptiles of which traces were found in their potential diet [55]. Numerous studies document the role of some of the detected species in AMR transmission [56–60]. The high proportion of tetracycline resistance found in foxes is probably related with oral vaccination of foxes against rabies since tetracycline is a vaccine absorption marker [61]. Another discovery was a surprisingly high AMR level to beta-lactams that we found in this species. The prevailing *bla*_OXA-347_ gene encoding class D beta-lactamase, the same as found in both poultry species, was previously noted in *Capnocytophaga stomatis* and *C*. *cynodegmi*, that constitute oral flora components of healthy cats and dogs [62]. Beta-lactam resistance was reported formerly in *E*.*coli* and enterococci isolated from red foxes [63, 64], but an important observation from our research was several genes encoding AmpC-type cephalosporinases being only found in this species. We assume that this result might be associated with tetracycline resistance, as genes encoding AmpC β-lactamases are often carried on plasmids along with other AMR genes including those determining tetracyclines resistance [65]. Finding that should be emphasized was the presence of AMR towards quinolones in red foxes. Previous studies confirmed quinolone resistance among *E*. *coli* and *Enterococcus* spp. isolated from this species but without indicating the specific AMR genes [64, 66]. The AMR mechanism we noted was dominated by efflux pumps encoded by the *oqx*A and *oqx*B genes. These pumps are disseminated on plasmids among *Enterobacteriaceae* and *Enterococci* [67–69]. Given the plasmid abundance found in this wild species and probability of *Salmonella* spp. occurrence, these findings are cause for concern, even taking into account the limitations of the method in plasmid analysis [70, 71].

The fact that we observed only traces of resistance in rodents compared to other animals should be underlined. The choice of this group of animals, collected at forest and meadow areas, was dictated by the fact that their probable contact with human settlements was occasional and therefore the animals had little contact with antimicrobials. Both the current and our previous results confirmed these assumptions [72]. Although insight into diet composition would indicate that rodents have invaded e.g. tobacco plantations. AMR found in rodents from urban areas or from regions with high livestock density seems to be more abundant [73, 74]. Studies on *E*. *coli* derived from rats from Hong Kong revealed high rate of ESBL producers in the rodents living in underground sewers [75]. Recent metagenomic analysis of urban sewage proved the broad diversity of AMR genes in that material [6].

### Bacterial and plasmid composition versus external factors–considerations for further studies

Insight into animal gut microbiomes and diet indicates that diverse dietary preferences result in differences in bacterial composition. A more varied diet seems to drive gut microbiome diversity, as in the case of wild animals. We could also expect higher diversity among resistance determinants found in wildlife. The contrary seems nevertheless to be the case; the variety of AMR genes appears to be greater in farm animals. The explanation for this might be significant exposure of those species to several drugs, disinfectants and metal ions e. g. copper used as feed additives [76]. The unexpectedly high abundance of plasmids in wild boars and red foxes captured our attention. We anticipated to detect more plasmids in domestic animals, the intestinal microbiomes of which theoretically had greater contact with antimicrobials. Though we are aware of the uncertain interpretation of these results, bearing in mind the limitations of the method in plasmid analysis [70, 71], still it is hard to ignore the result. Explanation of this is challenging. Bacteriome diversity and richness and contact with antimicrobial pressure may only partially explain the finding [77]. As plasmids constitute a bacterial tactic for adaptation to environmental changes, we presume that other external factors like xenobiotics, or a more varied diet might affect plasmid presence [78]. We hypothesize that in farm animals, the standard husbandry practices of feeding, treating disorders, and housing in closed farm environment might lead to selection of only a fraction of the bacterial flora or plasmids observed in an open natural environment. The high abundance of plasmids indicate the potential of wildlife as an AMR reservoir and transmission vector although more advanced analysis should be undertaken.

### The limitations and strengths of the study

To the best of the authors’ knowledge, this is the first study on AMR in animals that examined the possible diet of selected animals in the context of AMR spread.

Notwithstanding bias due to the limited number of samples could have been introduced in this study. The applied method has a limitation in plasmid analysis still, the obtained results were hard to ignore and the authors decided to include this data to indicate the considerations for further studies. Moreover, the overall quinolone and polymyxin resistance level described in this study might be underestimated due to the applied approach. The chromosomal mutations in the quinolone resistance determining region (QRDR) and mutations leading to colistin resistance might have been missed.

## Conclusion

Here, we applied metagenomics to investigate the epidemiology of AMR in various animal species. The study revealed higher AMR levels as well as higher resistome diversity and richness in domestic species, pointing to antimicrobial usage in the animal production sector as the main AMR driver. Our results also indicate that wildlife constitutes a reservoir of AMR determinants including those encoding resistance to antimicrobials highly important in human medicine. The potential of wildlife as AMR transmission vectors has been proven by plasmid profiles revealed in wild boars and red foxes. We also demonstrated that discrepancies between AMR found in the intestinal microbiomes of various animals probably resulted from different antimicrobial exposure, habitat, and diet. The overall results allowed us to highlight at least a few factors that may foster AMR spread in animals and clearly indicate anthropogenic impact on AMR dissemination.

## Supporting information

S1 Table Mapped summary. (XLSX)

S2 Table Raw and relative read abundances. (XLSX)
